# Expression of seven stem-cell-associated markers in human airway biopsy specimens obtained via fiberoptic bronchoscopy

**DOI:** 10.1186/1756-9966-32-28

**Published:** 2013-05-17

**Authors:** Laodong Li, Huina Yu, Xiaoyang Wang, Jinrong Zeng, Dangyu Li, Jingyan Lu, Changming Wang, Jiying Wang, Jianghong Wei, Ming Jiang, Biwen Mo

**Affiliations:** 1 Division of Respiratory Diseases, Guilin Medical University Hospital, Guilin, Guangxi, China; 2 VitroVivo Biotechnology, LLC, Rockville, MD, USA; 3 Division of Respiratory Diseases, Nan Xi Shan Hospital, Guilin Medical University Hospital, Guilin, Guangxi, China

**Keywords:** Stem-cell-associated marker, Lung cancer, Therapeutic target, Diagnostic marker

## Abstract

**Background:**

Previous reports have suggested that malignant transformations originate from adult stem cells, and may thus express the stem-cell-associated markers. The purpose of this study is to investigate the differential expression and clinical significance of seven stem-cell-associated markers (Bmi1, CD133, CD44, Sox2, Nanog, OCT4 and Msi2) in lung cancer, providing new targets for the diagnosis and treatment of lung cancer.

**Methods:**

In this study, we evaluated the differential expression of mRNA levels seven stem-cell-associated markers by semi-quantitative reverse transcription polymerase chain reaction (RT-PCR) from 112 human lung cancer and 18 non-cancer tissues obtained by bronchoscopy. We further verified the differential expression of these markers by immunohistochemistry in 50 lung cancer specimens, 30 benign inflammatory lesion tissues and 20 non-tumor adjacent lung tissues.

**Results:**

With the exception of OCT4, other markers Bmi1, CD133, CD44, Sox2, Nanog and Msi2 mRNA and protein were abundantly expressed in lung cancer. Additionally, Nanog expression was highly upregulated in lung cancer tissues and rarely presented in non-cancerous lung tissues, the sensitivity and specificity of Nanog mRNA reached 63.4% and 66.7%, respectively. Nanog therefore possessed high diagnostic value, however, CD44, Bmi1 and CD133 showed poor diagnostic value in lung cancer.

**Conclusion:**

Nanog may serve as a promising diagnostic marker of lung cancer and potential therapeutic target in lung cancer.

## Background

Lung cancer is one of the leading causes of cancer-related mortality in the world, and the incidence rates are increasing in many countries [1]. Although the prognosis is improving, the 5-year overall survival rate of lung cancer patients is still only approximately 16% [2]. In order to improve survival outcome, it is important to detect and surgically remove lung cancer at an early stage. Currently, the cancer stem cell (CSC) theory proposes that tumors contain a small subpopulation of CSC, which is responsible for tumor growth, invasion and metastasis [3]. CSC and normal tissue stem cells share important characteristics: self-renewal, multipotency and unlimited proliferation, and potentially overlapping molecular mechanisms [4,5]. In human adult tissues and tumors, several hundred stem-cell-associated markers have been identified. In lung cancer, the common stem-cell-associated markers include Bmi1, CD133, CD44, Sox2, OCT4 and so on [6,7]. Emerging evidences showed that these stem-cell-associated markers correlate with tumorigenesis, progression and metastasis, and may be as potential diagnostic markers for various human tumors [8-15].

Bmi1 is an oncogenic member of the polycomb group proteins involved in the self-renewal and differentiation of stem cells. The expression of Bmi1 mRNA has been shown to be a good marker to support the diagnosis of breast cancers in surgically resected specimens [8]. Likewise, CD133, a transmembrane glycoprotein which was first recognized in human hematopoietic stem cells, is considered the most representative marker to isolate CSC from lung cancer [9]. Recently, Moreira et al. [10] found CD133 is expressed in 58% of small cell lung cancers and 19% of lung adenocarcinomas, but not in normal lung tissue, suggesting that CD133 could be used as a potential diagnostic marker in lung cancer. Another surface marker, CD44, has also been used to isolate CSC from lung cancer [11]. A previous study using competitive RT-PCR to detect the expression of CD44 in urine for bladder cancer diagnosis was highly accurate and a potential non-invasive diagnostic marker for bladder cancer [12]. Transcription factors, Sox2, OCT4 and Nanog form a core regulatory network of self-renewal and differentiation in embryonic stem cells, which are essential in sustaining stem cell pluripotency [13]. Recent reports show that Sox2, OCT4 and Nanog are potential diagnostic markers for lung cancer [14-16]. Additionally, Musashi2 (Msi2), a RNA binding protein, play crucial roles in maintaining self-renewal and pluriopentency of embryonic stem cells. It have been demonstrated to participate in tumorigenesis and progression of multiple solid tumors [17,18], and are expressed in lung cancer [10]. However, these studies which are mainly based on surgical specimens to screen for new molecular markers have certain limitations in clinical application because most lung cancers are unresectable.

Bronchoscopy has become an essential method by which to analyze and diagnose lung cancer through technological advances and its widespread application. Common bronchoscopy techniques including forceps biopsy, brushing and washing can easily obtained adequate specimens for histological, cytological and molecular biological analysis [19]. The purpose of this study is to investigate the differential and clinical significance of these stem-cell-associated markers in bronchoscopy biopsy specimens.

In this study, we applied RT-PCR to examine the differential expression of Bmi1, CD133, CD44, Sox2, Nanog, OCT4 and Msi2 mRNA in bronchoscopic biopsy specimens from lung cancer and non-cancer patients. Furthermore immunohistochemistry was used to define the localization and expression patterns of these stem-cell-associated proteins in surgically resected lung cancer and non-malignant lung tissues. The diagnostic value of these seven stem-cell-associated markers was evaluated in lung cancer.

## Materials and methods

### Clinical samples from bronchoscope biopsy

This prospective study in 112 patients with histologically proven lung cancer and 18 non-cancer patients was performed at Guilin Medical University Hospital and Affiliated Nan Xi Shan Hospital in China from January, 2011 to January, 2012. These 112 lung cancer patients included 94 males and 18 females ranging from 29 to 80 years of age (median = 59.2). Fifty-six cases were squamous cell carcinomas (SCC), 17 cases adenocarcinomas (Ad), 28 cases small cell lung carcinomas (SCLC) and 11 cases of other types of lung cancer. Based on clinical and radiological findings, 100 cases had been evaluated for stages: 7 cases of stage I, 6 cases of stage II, 60 cases of stage III and 27 cases of stage IV of lung cancer. Among 18 cases of non-cancer, 7 cases were bronchitis, 7 cases tuberculosis, 3 cases pneumonia and 1 case brochiectasis. All patients had not received any anti-cancer therapy before receiving bronchoscopy. At least 5 biopsy specimens were obtained from one patient. One to two specimens were snap frozen and stored at -80°C for RT-PCR analysis under the condition of specimens were sufficient for routine diagnosis. The remaining specimens were fixed in buffered formalin for histopathological evaluation. This study was approved by the Guilin Medical University Review Board, and informed consent was obtained from all patients under the protocols prescribed by the Guilin University Ethics Committee.

### Semi-quantitative RT-PCR

Total RNA was isolated from the biopsy tissue using Trizol reagent (TakaRa Bio Inc, Dalian, China) according to the manufacturer's instructions. One μg of the mRNA was reverse transcribed to cDNA using PrimeScript II 1st Strand cDNA Synthesis Kit (TakaRa). One μl of the cDNA was used in PCR for the amplification of β-actin and seven stem-cell-associated markers. The primers are presented in Table 1. The DNA thermal cycler conditions used were 94°C for 5 min (pre-denature), and 35 cycles of 94°C for 1 min, annealing for 30 s and extension at 72°C for 45 s, followed by a final extension of 72°C for 2 min. Six μl of each PCR-amplified product were separated on a 2% agarose gel, which was then visualized by ethidium bromide staining using a JS-780 Gel Image Analysis System (Peiqing Sci Tech, Ltd, Shanghai, China). The ratio of integrated density of target genes over corresponding β-actin was normalized as relative mRNA expression levels of stem-cell-associated markers.

**Table 1:** The primers and primary antibody used in this study

**Gene symbles**	**Primers for RT-PCR**			**Antibodies for IHC**		
		**Primer sequences**	**Annealing temperature (°C)**	**Antibody sources**	**Clone**	**Dilution**
Bmi1	Reverse	5’-ATT GTC TTT TCC GCC CGC TT-3’	58.2	ProMab Biotechnologies Inc	3E3	1:800
	Forward	5’-TGG CAT CAA TGA AGT ACC CTC-3’				
CD44	Reverse	5’-TGC TAC TGA TTG TTT CAT TGC G-3’	56.2	ProMab Biotechnologies Inc	8E2F3	1:30000
	Forward	5’-GGA CCA GGC CCT ATT AAC CC-3’				
CD133	Reverse	5’-AAA CAA TTC ACC AGC AAC GAG-3’	54.1	ProMab Biotechnologies Inc	3 F10	1:400
	Forward	5’-TAG TAC TTA GCC AGT TTT ACC G-3’				
Sox2	Reverse	5’- GCT AGT CTC CAA GCG ACG AA-3’	56.2	ProMab Biotechnologies Inc	10 F10	1:800
	Forward	5’- TAC AGT CTA AAA CTT TTG CCC TT-3’				
Nanog	Reverse	5’-AGG CAA CTC ACT TTA TCC CAA-3’	54.1	Cell signaling technology	D73G4	1:300
	Forward	5’-GAT TCT TTA CAG TCG GAT GCT T-3’				
Oct-4	Reverse	5’-TGC AGA AAG AAC TCG AGC AA-3’	56.2	Santa Cruz Biotechnology	C-10	1:50
	Forward	5’-CTC ACT CGG TTC TCG ATA CTG G-3’				
Msi2	Reverse	5’-CAG ACC TCA CCA GAT AGC CTT-3’	56.2	ProMab Biotechnologies Inc	2C11	1:1000
	Forward	5’-TAC TGT GTT CGC AGA TAA CCC-3’				
β-actin (217 bp)	Reverse	5’GTG ACG TGG ACA TCC GCA AAG-3’	60.2			
	Forward	5’-ATC CAC ATC TGC TGG AAG GTG GAC-3’				
β-actin (417 bp)	Reverse	5’-ACA GAG CCT CGC CTT TGC CGA TC-3’	60.2			
	Forward	5’-TGG GTC ATC TTC TCG CGG TTG G-3’				

### Immunohistochemistry (IHC)

A total of 50 cases of surgically resected lung cancer, 30 benign inflammatory lesion tissues and 20 normal or non-tumor adjacent lung tissues were used for IHC experiments. The lung cancer samples consisted of 17 adenocarcinomas (Ad), 3 bronchioloalveolar carcinomas (BAC), 23 squamous cell carcinomas (SCC) and 7 small cell lung carcinomas (SCLC). Thirty cases of benign inflammatory lesion samples included 12 cases of tuberculosis, 6 cases of pneumonia, 6 cases of inflammatory pseudotumor, 3 cases of brochiectasis, 2 cases of lung abscess and 1 case of benign fibroma of lung. In 50 non-cancer lung tissues 3 cases were squamous metaplasia including 2 cases of non-tumor adjacent lung tissues and 1 case of pneumonia. In all patients bronchoscopy and surgery were performed at Guilin Medical University Hospital from January, 2002 to December, 2011. None of the subjects received radiation therapy or chemotherapy before surgery.

Surgical specimens were fixed in 10% formaldehyde, and paraffin-embedded. After deparaffinization and rehydration, the 4 μm sections underwent antigen retrieval by boiling in 10 mM citrate buffer (pH 6.0) or EDTA (pH 8.0). The sections were immersed in H_2_O_2_ for 10 min and washed with PBS three times. Then the sections were incubated for 1 hr with the primary antibodies (Table 1) at 37°C. After a brief wash, the sections were incubated for 20 min with Polymer Helper (ZSGB-BIO, Beijing, China). Sections were washed three times with PBS and the antigen was visualized with polyperoxidase-anti-mouse/rabbit IgG (ZSGB-BIO) and DAB as substrate (ZSGB-BIO). The sections were counterstained with Mayer hematoxylin and mounted in Permount. Blank controls were obtained by replacing the primary antibodies with PBS.

The expression pattern criteria determined by IHC included: ‘diffuse’ when almost all cells expressed the antigen; ‘focal’ when isolated groups of positive cells were seen within a histological section; ‘isolated staining’ when single cells were positive for the marker. All slides were reviewed by a pathologist (Lu JY, Guilin, China) and a well-trained researcher in pathology (Li LD, Guilin, China) blinded to the patients' clinical information.

### Statistical analysis

The Chi-Square test and the Mann–Whitney *U* test were applied to compare the expression of markers between lung cancer and non-cancer. The Chi-Square test was also performed to analyze the association between mRNA expression markers and lung cancer clinical factors. For evaluation of diagnostic value of seven stem-cell-associated markers mRNA, the following calculations were made: sensitivity (%) was calculated as true-positive/(true-positive +false-negative)×100, specificity (%) as true-negative/(true-negative + false-positive)×100 and diagnostic accuracy (%) as (true-positive + true-negative/(true-positive + false-negative + false-positive + true-negative)×100, where true-positive denotes specimens with stem-cell-associated marker positive expression and lung cancer diagnosis during follow up, true-negative denotes negative expression with no lung cancer, false-positive denotes positive expression with no lung cancer and false-negative denotes negative expression and diagnosed lung cancer. All statistical analyses were performed by SPSS 17.0 software package for Windows. P<0.05 was regarded statistically significant.

## Results

### The mRNA expression of seven stem-cell-associated markers in biopsy samples obtained through bronchoscopy

**Figure 1:**
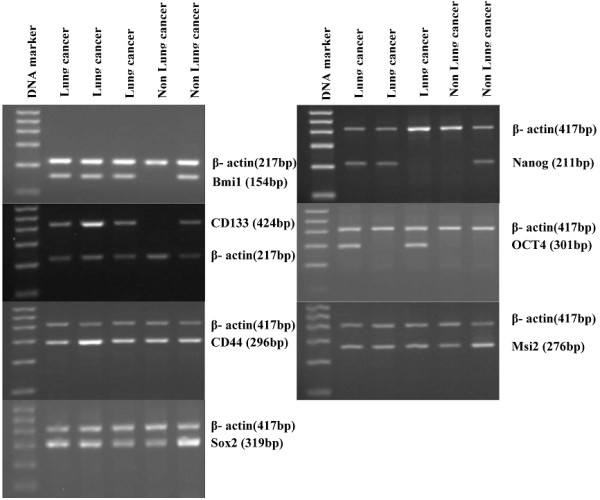
**Example RT-PCR bands of human lung cancer and non-lung cancer biopsy tissues obtained from bronchoscopy.** Total RNAs were isolated and reverse transcribed to cDNA from the biopsy tissues. RT-PCR Products of β-actin and stem-cell-associated markers were run on 2% agarose gels with ethidium bromide.

The expression of Bmi1, CD133, CD44, Sox2, Nanog, OCT4 and Msi2 mRNA in bronchoscopic biopsies of lung cancer and non-cancer patients are presented in Table 2 and Figure 1. Overall, the mRNA expression of seven markers was higher in the malignant group than in the benign group. However, the mRNA relative levels of Bmi1, CD133 and CD44 by RT-PCR were not significantly different between lung cancer and non-malignant lung tissues analyzed by Mann–Whitney *U* test, nor were the expression rates of CD44 and Msi2. We found that the Bmi1 positive expression rate was significantly correlated with histology types (P=0.007) and differentiation (P=0.027), while the positive rate of Nanog was negatively correlated with differentiation (0.032). However, the positive expression rates of CD133, CD44, Sox2, OCT4 and Msi2 did not correlate with age, gender, histological type, stage and differentiation of lung cancer (Table 3).

**Table 2:** mRNA expression of stem cell makers in human lung cancer and non-cancer lung tissues

	**Lung cancer**	**Non-cancer**	**P**	**Lung cancer**	**Non-cancer**	**P**
	**Positive rate, %(n)**	**Positive rate, %(n)**		**Expression, χ ± s**	**Expression, χ ± s**	**Value**
Bmi1	88.4(99/112)	66.7(12/18)	0.026	0.60±0.73	0.32±0.29	0.118
CD133	85.7(96/112)	55.6(10/18)	0.006	0.77±0.90	0.58±0.97	0.057
CD44	98.2(110/112)	88.9(16/18)	0.092	1.67±1.77	1.44±1.33	0.606
Sox2	98.2(110/112)	83.3(15/18)	0.019	2.06±2.15	0.99±1.53	0.001
Nanog	63.4(71/112)	33.3(6/18)	0.016	0.23±0.42	0.04±0.09	0.013
OCT4	85.7(96/112)	38.8(7/18)	<0.0001	0.46±0.50	0.12±0.27	<0.0001
Msi2	96.4(108/112)	94.4(17/18)	0.531	1.29±1.13	0.47±0.51	<0.0001

**Table 3:** Correlation between stem cell mRNA expression of biopsy samples and lung cancer clinical features

	**Analyzable**	**Bmi1 expression**	P*	**CD133 expression**	P*	**CD44 expression**	P*	**Sox2 expression**	P*	**Nanog expression**	P*	**OCT4 expression**	P*	**MSi2 expression**	P*
	**cases**	**Postive, n(%)**		**Postive, n(%)**		**Postive, n(%)**		**Postive, n(%)**		**Postive, n(%)**		**Postive, n(%)**		**Postive, n(%)**	
Age															
<60	57	51(89.5)	0.716	48(84.2)	0.643	56(98.2)	1	55(96.5)	0.496	36(63.2)	0.958	47(82.5)	0.448	54(94.7)	0.618
≥60	55	48(87.3)		48(87.3)		54(98.2)		55(100)		35(63.6)		49(89.1)		54(98.2)	
Gender															
Male	94	84(89.4)	0.436	80(85.1)	1	92(97.9)	1	92(97.9)	1	57(60.6)	0.167	79(84.0)	0.462	90(95.7)	1
Female	18	15(83.3)		16(88.9)		18(100)		18(100)		14(77.8)		17(94.4)		18(100)	
Histology															
SCLC	28	27(96.4)	0.007	27(96.4)	0.066	26(92.9)	0.171	27(96.4)	1	22(78.6)	0.068	26(92.9)	0.601	27(96.4)	1
Ad	17	11(64.7)		16(94.1)		17(100)		17(100)		13(76.5)		14(82.4)		16(94.1)	
SCC	56	52(92.9)		43(76.8)		56(100)		55(98.2)		31(55.4)		46(82.1)		54(96.4)	
other	11	9(81.8)		10(90.9)		11(100)		11(100)		5(45.5)		10(90.9)		11(100)	
Stage															
I~II	13	13(100)	0.601	11(84.6)	1	13(100)	1	13(100)	1	7(53.8)	0.369	10(76.9)	0.407	13(100)	1
III~IV	87	78(89.7)		74(85.1)		85(97.7)		85(97.7)		58(66.7)		75(86.2)		84(96.6)	
Differentiation															
Well-Moderate	28	21(75)	0.027	21(75)	0.216	28(100)	1	27(96.4)	0.337	12(42.9)	0.032	22(78.6)	0.537	26(92.9)	0.262
Poor	55	52(94.5)		48(87.3)		55(100)		55(100)		37(67.3)		47(85.5)		54(96.4)	

* chi-square test . Ad = adenocarcinomas, SCC = squamous cell carcinomas, SCLC = small cell lung carcinomas and others = mucoepidermoid carcinoma, malignant mesothelioma or unclarified lung cancer.

### Localization and expression patterns of stem-cell-associated markers protein in non-malignant lung tissues and lung cancer

Based on our RT-PCR results, most of the stem-cell-associated markers mRNA were expressed in non-malignant lung tissues although the expression levels were relative low. Therefore, we further examined the localization and expression patterns of stem cell markers in non-malignant lung tissues and lung cancer by IHC.

**Figure 2:**
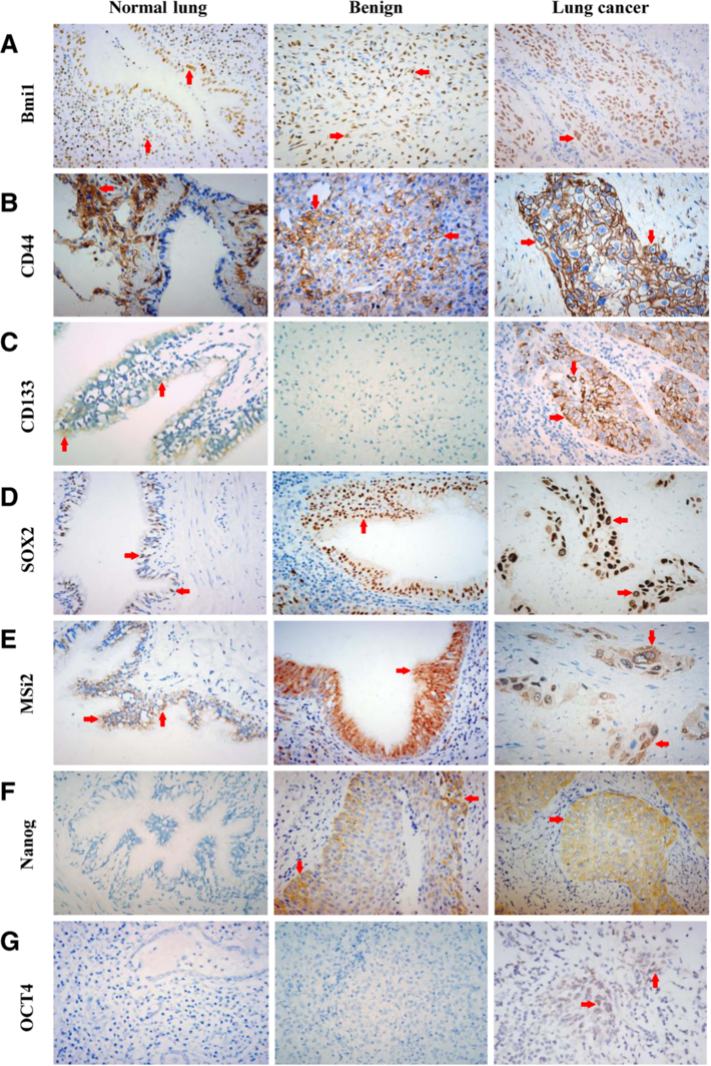
**Representative the expression of Bmi1, CD44, CD133, Sox2, Nanog, OCT4 and Msi2 in normal lung, benign lesion and lung cancer.** (**A**) Nuclear staining of Bmi1 is expressed (red arrows) in normal lung, benign fibroma of lung and squamous cell carcinomas. (**B**) Membranous staining of CD44 in normal lung, benign fibroma of lung and squamous cell carcinomas. (**C**) Membranous and cytoplastic staining of CD133 in normal bronchial epithelium cells and squamous cell carcinomas, negative immunostaining signal of CD133 in benign fibroma of lung. (**D**) Nuclear staining of Sox2 in normal bronchial epithelium cells, squamous metaplasia and squamous cell carcinomas. (**E**) Cytoplastic and nuclear staining of Msi2 in normal bronchial epithelium cells, squamous metaplasia and squamous cell carcinomas. (**F**) Negative immunostaining signal of Nanog in normal lung, cytoplastic staining of Nanog in squamous metaplasia and squamous cell carcinomas. (**G**). Negative immunostaining signal of OCT4 in normal lung and tuberculosis, nuclear staining of OCT4 in small cell lung carcinomas. All images were taken at 400× magnification.

Bmi1 was diffusely expressed in bronchial epithelium cells, alveolar epithelium cells, lung interstitial cells and some inflammatory cells of all non-malignant lung tissues (Figure 2A), and was diffusely expressed in 47 cases of lung cancer and focally expressed in 1 case of Ad and 1 case of SCLC (Figure 2A). Similar to Bmi1, CD44 was abundantly expressed in alveolar epithelium cells, lung interstitial cells, macrophages, inflammatory cells and metaplastic squamous bronchial epithelium of non-malignant lung tissues (Figure 2B), but was absent in normal bronchial epithelium cells. 38 out of 50 lung cancer tissues were positive for CD44, of which 37 cases were diffusely positive and 1 case was focally positive expression (Figure 2B).

In non-malignant lung tissues, CD133 was exclusively expressed in some, but not all, bronchial epithelium cells and bronchial smooth muscle cells (Figure 2C). CD133+ bronchial epithelium cells were found in 74% of non-malignant lung tissues while CD133+ bronchial smooth muscle cells were 70%. In lung cancer tissues, about 56% of tumor samples were diffusely positive, 8% focally positive and 2% isolated positive for CD133 (Figure 2C).

In non-malignant lung tissues, all bronchial epithelium and squamous metaplasia showed positive expression of Sox2 (Figure 2D) and Msi2 (Figure 2E), the expression decreases in terminal bronchioles and was absent in alveolar epithelial. In lung cancer, the expression of Sox2 and Msi2 was 90% and 94% respectively, and more than 85% of tissues was diffusely positive for both of the markers (Figure 2D, E).

In non-malignant lung tissues, only 2 cases of squamous metaplasia in non-tumor adjacent lung tissues were positive for Nanog (Figure 2F), whereas, Nanog staining was detected in 36 of 50 (72%) cases of lung cancer, in which 29 cases were diffusely positive, 6 cases were focally positive and 1 case was isolated positive (Figure 2F).

In all non-malignant lung tissues, no positivity for OCT4 was observed (Figure 2G). In lung cancer group, only one case of SCC and one case of SCLC were focally positive for OCT4 (Figure 2G).

### Potential value of the expression of stem-cell-associated markers as diagnostic markers

Table 4 describes the specificity, accuracy and sensitivity of seven stem-cell-associated markers mRNA in bronchoscopic biopsies of lung cancer and non-cancer patients. The stem-cell-associated markers with the highest sensitivity for malignancy were CD44 (98.2%), Sox2 (98.2%) and Msi2 (96.4%), but their specificity were too low to be considered of no clinical significance. Nanog exhibited the highest specificity which was 66.7%, and its sensitivity was 63.4%.

**Table 4:** The specificity, accuracy and sensitivity of seven stem-cell-associated markers mRNA in biopsy samples obtained from bronchoscopy

	**Specificity, %**	**Accuracy, %**	**Sensitivity, %**
Bmi1	33.3	80.8	88.4
CD133	44.4	80	85.7
CD44	11.1	86.2	98.2
Sox2	16.7	86.9	98.2
Nanog	66.7	63.8	63.4
OCT4	61.2	82.3	85.7
Msi2	5.6	83.8	96.4

## Discussion

In the present study, we evaluated the differential expression of seven stem-cell-associated markers in bronchoscopic biopsies of lung cancer and non-cancer patients by RT-PCR, and assessed their diagnostic value potential. Our data found Nanog mRNA had the highest specificity in lung cancer. We further confirmed the high diagnostic value of Nanog protein levels by IHC, Nanog was overexpressed in lung cancer tissues, but rarely expressed in non-malignant lung tissue. Taken together, these results demonstrate that Nanog mRNA is a potential diagnostic marker for lung cancer.

Nanog is a transcription factor that plays an important role in maintaining self-renewal of embryonic stem cells. Current studies have reported that the expression of Nanog was higher in multiple cancerous tissues than in their normal counterparts, including breast cancer [20], gastric adenocarcinomas [21], colorectal cancer [22], gliomas [23] and ovarian serous cystadenocarcinomas [24]. In this study, we found the expression of Nanog mRNA in bronchoscopic biopsies of lung cancer patients was significantly higher compared to that in non-cancer patients. Although Nirasawa et al. [16] have also reported that the expression of Nanog mRNA was higher in surgically resected lung cancer tissues than in non-cancerous tissues, it is not known what cells express Nanog in non-cancerous lung tissues. Using IHC, we found Nanog was only expressed in metaplastic squamous bronchial epithelium cells in 2 out of 50 non-malignant lung tissues, and was negative in normal airway epithelia. Therefore, Nanog may be a good diagnostic marker for lung cancer.

In this study, our results showed that the mRNA levels of Bmi1, CD44 and CD133 were not significantly different between lung cancer and non-malignant lung tissues. Further analyzed by IHC, we observed that Bmi1, CD44 and CD133 were not only expressed in lung cancers, Bmi1 and CD44 were also abundantly expressed in lung interstitial cells, inflammatory cells and bronchial epithelium cells, and CD133 was diffusely expressed in some normal bronchial epithelium cells and bronchial smooth muscle cells, consistent with previous studies [11,25,26]. Hence, Bmi1, CD44 and CD133 are poor diagnostic markers for lung cancer.

Likewise, although the expression levels of Sox2 and Msi2 mRNA in lung cancer tissues were significantly higher as compared with non-malignant tissues, we found more than 80% of bronchoscopic biopsy specimens of non-cancer patients were positive for Sox2 and Msi2 mRNA, and all non-malignant tissues were positive for Sox2 and Msi2 protein expression, consistent with previous findings [10,27,28]. Therefore, Sox2 and Msi2 have poor diagnostic specificity in lung cancer.

It is still controversial whether lung cancer cells express OCT4. Some researchers believe that OCT4 is involved in tumorigenesis and metastasis of lung cancer, and therefore is a potential diagnostic marker and useful therapeutic target of lung cancer [9,15,29], while others did not detect OCT4 expression in lung cancer [10,30]. Although we observed OCT4 mRNA expression in 85.7% of lung cancer and 38.8% of non-cancer bronchoscopic biopsy specimens, but OCT4 protein was nearly absent in 50 cases of lung cancer tissues. The reason for this discrepancy is unclear, but may be due to complex mechanism of post-transcriptional regulation, or potential presence of unknown OCT4 pseudogenes which cause false positive detection by RT-PCR. Therefore, the diagnostic value of OCT4 mRNA in bronchoscopic biopsy specimens requires further investigation.

In addition, we examined the correlation of seven stem cell markers expression in bronchoscopic biopsy specimens of lung cancer with patient clinical features. As we know, poorly differentiated cancers show stronger aggressive and metastatic ability [21]. We found the positive expression rates of Nanog and Bmi1 mRNA was inversely correlated to differentiation of lung cancer, indicating these two markers may be useful to predict tumor progression and poor prognosis in lung cancer. Chiou et al. [29] reported that Nanog expression in surgically resected lung cancer tissues is an independent prognostic factors of poor prognosis for patients. Vrzalikova and colleagues [31] also believed that the expression of Bmi1 in surgically resected lung cancer tissues is a prognostic marker in lung cancer. However, surgical resection is not an option for all lung cancer patients, and therefore the use of these markers in bronchoscopic biopsies to predict prognosis would be a great clinical advantage.

## Conclusions

In conclusion, the expression of Nanog mRNA in bronchoscopic biopsy specimens is useful diagnostic marker for lung cancer. Further investigation of the diagnostic potential of Nanog in early stages of lung cancer may have a profound clinical impact.

## Competing interests

The authors declare that they have no competing interests.

## Authors’ contributions

LDL and HNY collected data and specimens, carried out the RT-PCR and immunochemistry staining, analyzed the results and drafted the manuscript. XYW conceived and designed the experiments, drafted and revised the manuscript critically and gave final approval of the version to be published. JRZ and DYL helped to collected bronchoscopic biopsy specimens. JYL helped to carry out the immunochemistry staining and assessed the slides. CMW, JYW, JHW and MJ participated in study coordination and statistical analysis. BWM conceived and designed of the study, performed the interpretation of data, literature search, writing and revising. All authors read and approved the final manuscript.
